# Excellent response to high-dose Riboflavin in Brown-Vialetto-Van Laere syndrome: A case report

**DOI:** 10.1097/MD.0000000000051027

**Published:** 2026-10-02

**Authors:** Aasim Ali, Armaghana Abdullah, Hafiz Muhammad Yousaf Masood, Usama Saleem, Muhammad Talha, Labeeba Abdul Ghafoor, Mukesh Kumar Sharma

**Affiliations:** a Department of Neurology, Allied Hospital Faisalabad, Faisalabad, Pakistan; b Department of Internal Medicine, Allied Hospital Faisalabad, Faisalabad, Pakistan; c Department of Neurology, Dhankuta Multiple Campus, Tribhuvan University, Nepal.

**Keywords:** brown, dose riboflavin, high, riboflavin transporter deficiency, *SLC52A3*, Van Laere syndrome, vialetto

## Abstract

**Rationale:**

Brown-Vialetto-Van Laere syndrome, currently recognized within the spectrum of riboflavin transporter deficiency type 3 (RTD3), is a rare inherited neurometabolic disorder associated with pathogenic variants in *SLC52A3*. It typically presents with progressive sensorineural hearing loss, pontobulbar dysfunction, peripheral neuropathy, and respiratory involvement. Diagnosis may be delayed because routine metabolic and neuromuscular investigations can be unrevealing despite a characteristic clinical phenotype.

**Patient concerns:**

A young Asian man presented with several years of progressive bilateral hearing loss followed by worsening dysphagia to solids and liquids, symmetrical limb weakness, muscle wasting, and acute-on-chronic dyspnea. Two siblings had previously died at a young age after developing a similar sequence of hearing impairment, dysphagia, limb weakness, and unexplained respiratory failure.

**Diagnoses:**

Neurological examination showed tongue atrophy with fasciculations, thenar wasting, bifacial weakness, mild ptosis, diminished gag reflex, and symmetrical limb weakness. Audiometry confirmed profound bilateral sensorineural hearing loss. Nerve conduction studies demonstrated predominantly motor axonal neuropathy, while electromyography showed chronic and active denervation. Repetitive nerve stimulation, cerebrospinal fluid analysis, magnetic resonance imaging, creatine kinase levels, and metabolic investigations were unrevealing. Targeted genetic testing detected pathogenic SLC52A3 variants, confirming RTD3/ Brown-Vialetto-Van Laere syndrome.

**Interventions:**

High-dose oral riboflavin was initiated empirically at 40 mg/kg/day in 3 divided doses before genetic confirmation because of progressive respiratory compromise and strong clinical suspicion. Supportive management included nutritional support and nocturnal noninvasive ventilation with bilevel positive airway pressure.

**Outcomes:**

The patient showed marked improvement after riboflavin initiation. Respiratory effort improved within several days, and forced vital capacity increased from 35% to 50% of predicted within 1 week. Limb strength improved, and by 8 weeks the patient had gained 3 kg with stabilization of dysphagia and improved oral intake. At 6 months, neurological status remained stable without further electrophysiological progression. Riboflavin was well tolerated.

**Lessons:**

RTD3 should be considered in young patients with progressive sensorineural hearing loss associated with bulbar dysfunction, motor neuropathy, or respiratory weakness, particularly when family history is suggestive. Recognition of the characteristic phenotype, targeted genetic testing, and prompt initiation of high-dose riboflavin may substantially alter the clinical course of this potentially treatable disorder.

## 1. Introduction

Brown-Vialetto-Van Laere (BVVL) syndrome, now more precisely classified within the spectrum of riboflavin transporter deficiency type 3 (RTD3), is a rare autosomal recessive neurometabolic disorder caused by pathogenic variants in the *SLC52A3* gene, which encodes the riboflavin transporter RFVT3.^[1,2]^ Impaired riboflavin transport disrupts intracellular availability of flavin adenine dinucleotide and flavin mononucleotide, essential cofactors for multiple enzymatic pathways involved in mitochondrial energy metabolism, fatty-acid oxidation, and cellular redox reactions.^[2,3]^ Clinically, RTD3 is characterized by variable combinations of progressive sensorineural hearing loss, pontobulbar dysfunction, motor or sensorimotor neuropathy, muscle weakness, and respiratory involvement.^[1–3]^ Respiratory muscle weakness may become life-threatening, while substantial phenotypic overlap with motor neuron disorders, mitochondrial disease, and other hereditary neuropathies can delay recognition of this potentially treatable condition.^[1,4]^

What makes the present case particularly informative is the prolonged diagnostic course despite a highly suggestive clinical phenotype and a striking family history of 2 siblings who died following a similar sequence of hearing loss, bulbar dysfunction, weakness, and respiratory failure. Extensive conventional metabolic, neuromuscular, and genetic investigations were unrevealing before targeted testing for riboflavin transporter deficiency was undertaken. The case therefore illustrates the importance of recognizing the characteristic clinical pattern of RTD3 and maintaining diagnostic suspicion even when routine investigations are normal. Early recognition is especially important because high-dose riboflavin treatment can substantially modify the clinical course, and treatment should not necessarily be delayed when the phenotype is strongly suggestive and progressive respiratory involvement is present.^[1,2]^

## 2. Patient information

The patient is a young Asian male in his late teens or early twenties. He presented to a tertiary neurology center with multiple, progressive concerns. His primary symptoms were a gradual loss of hearing affecting both ears over the last few years, and a more recent, subacute progression of severe dysphagia. Over a period of several months, the dysphagia had worsened from an initial difficulty with solid foods to including liquids, causing frequent choking episodes and significant, unintentional weight loss. He also reported a mild, symmetrical weakness in his hands and feet, manifesting as difficulty with fine motor tasks like buttoning clothes and a tendency to trip while walking. In the few days immediately before admission, he developed progressively worsening shortness of breath, which was initially exertional but rapidly became present at rest, accompanied by orthopnea. He denied any diplopia, sensory loss, or bowel and bladder disturbances (Table 1 shows timeframe of clinical events).

**Table 1:** Timeline of clinical events.

Timeframe	Clinical event
~4 to 5 yr prior	Insidious onset of bilateral progressive hearing loss, initially noticed as difficulty understanding conversations in noisy environments.
~6 mo prior	Onset of progressive dysphagia, initially affecting solid foods, accompanied by mild weight loss.
~3 m oprior	Dysphagia progressed to involve liquids. Mild distal weakness of the hands and feet became apparent, and the patient became increasingly socially withdrawn.
~10 d prior	Development of exertional shortness of breath, which progressively worsened over the following days.
D 1 (presentation)	Admitted with acute-on-chronic dyspnea at rest. Neurological examination revealed tongue and thenar atrophy, bifacial weakness, and a diminished gag reflex. Nerve conduction studies (NCS) demonstrated an axonal neuropathy.
D 2 to 3	Further diagnostic evaluation was performed. Cerebrospinal fluid (CSF) analysis and repetitive nerve stimulation (RNS) were normal. Genetic panels for mitochondrial disorders and hereditary myopathies/dystrophies were sent, with results pending.
D 5	Strong clinical suspicion for Brown-Vialetto-Van Laere (BVVL) syndrome was established based on the characteristic phenotype and family history. Empiric high-dose oral riboflavin (40 mg/kg/day) was initiated while awaiting genetic confirmation. Targeted *SLC52A2/SLC52A3* sequencing was sent.
D10	Marked subjective and objective improvement in limb strength and respiratory effort was observed following initiation of riboflavin therapy.
Wk 4	Genetic testing confirmed biallelic pathogenic *SLC52A3* variants, establishing the diagnosis of BVVL syndrome/riboflavin transporter deficiency type 3.
Wk 8 (Outpatient follow-up)	Sustained clinical improvement was observed, with weight gain and stabilization of neurological symptoms. Repeat NCS demonstrated no further progression of axonal loss.
Mo 6	Neurological status remained stable. The patient was independently ambulatory, tolerated a modified soft oral diet, and required only nocturnal bilevel positive airway pressure (BiPAP) support.

The patient’s family history was the most critical alarm signal. He was the third of 5 siblings. Two of his siblings a brother and a sister had died in their early twenties several years prior after experiencing a nearly identical sequence of symptoms: insidious hearing loss in adolescence, followed by progressive swallowing difficulties, limb weakness, and ultimately, fatal respiratory failure of an unknown cause. No definitive diagnosis had been established for either sibling despite investigations at the time. The patient’s parents are clinically unaffected. There is history of consanguinity and the family originates from a small, relatively isolated geographic region in South Asia. The patient’s psychosocial situation was marked by profound anxiety and depression related to the loss of his siblings and the terrifying realization that he was developing the same, apparently untreatable, disease. Prior to the correct diagnosis, no specific therapeutic interventions had been attempted, and his condition was managed with only supportive and nutritional measures, which had yielded no improvement.

## 3. Clinical findings

On general physical examination, the patient was notably cachectic with visible muscle wasting and a body mass index below the 5th percentile for his age. His respiratory rate was 28 breaths per minute at rest with shallow tidal volumes, and use of accessory respiratory muscles and mild abdominal paradox were noted, signaling diaphragmatic weakness and impending ventilatory failure. Cranial nerve examination was significant for several findings. He had a poor, transverse smile due to bilateral facial muscle weakness and mild, symmetrical ptosis without ophthalmoplegia. The most pathognomonic finding was advanced tongue atrophy (Fig. 1), where the tongue appeared shrunken, furrowed, and exhibited continuous, worm-like fasciculations at rest. The gag reflex was markedly diminished upon bilateral pharyngeal stimulation. Motor examination of the limbs revealed symmetrical wasting of the intrinsic hand muscles, specifically pronounced thenar atrophy (Fig. 2) with relative sparing of the hypothenar eminences in a split-hand like appearance. There was also atrophy of the anterior compartment of the lower legs. Muscle strength, as graded by the Medical Research Council scale, was 4/5 in all 4 limbs proximally and 4 to 5 distally involving both upper and lower extremities. Deep tendon reflexes were globally depressed. Plantar responses were flexor, and sensory examination showed mildly decreased pain and temperature sensation distally.

**Figure 1.**
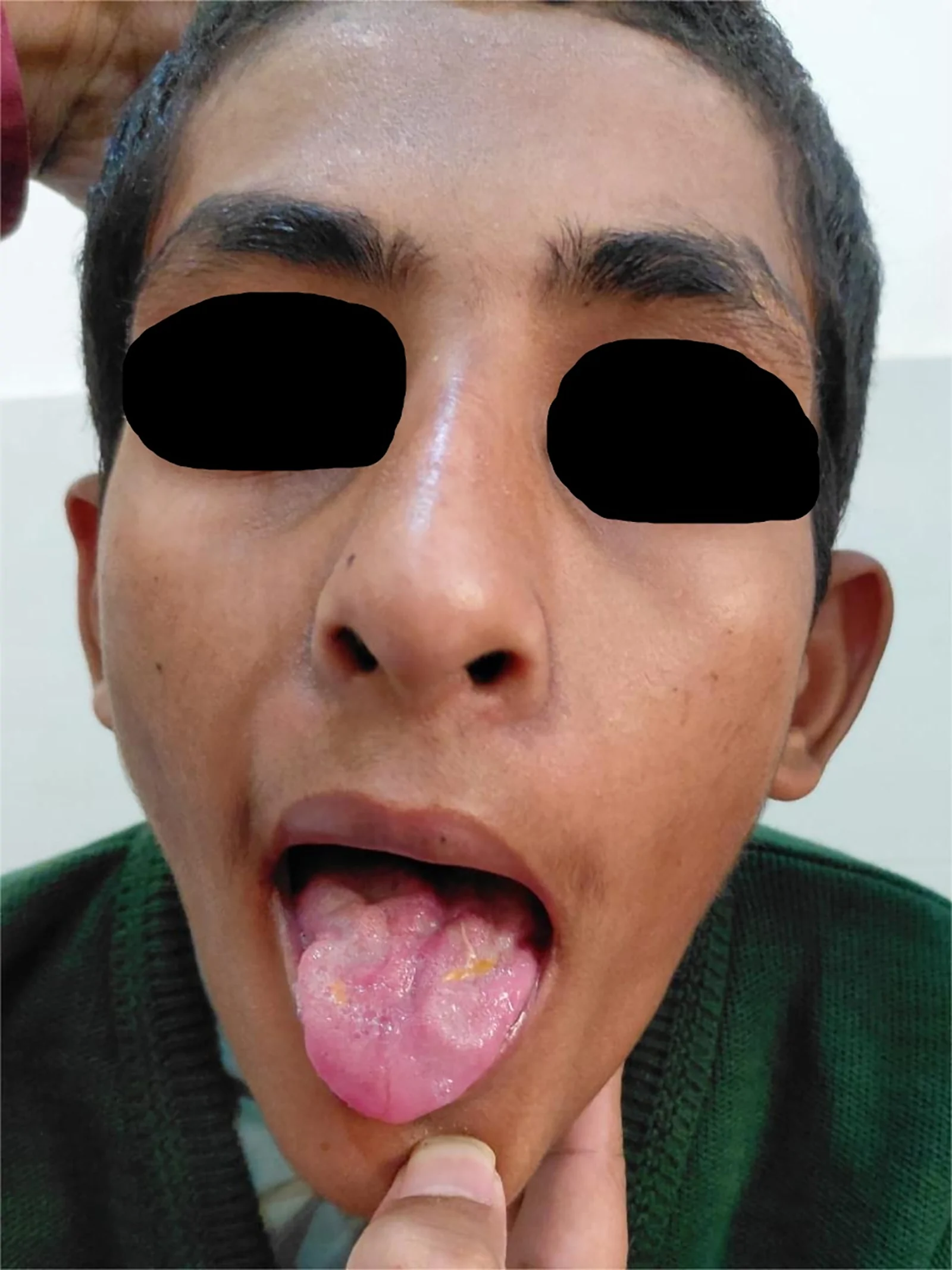
Clinical photograph demonstrating marked tongue atrophy. Clinical photograph of the oral cavity showing marked tongue atrophy with a furrowed and irregular surface. Tongue fasciculations were also observed during bedside neurological examination, consistent with bulbar motor involvement.

**Figure 2.**
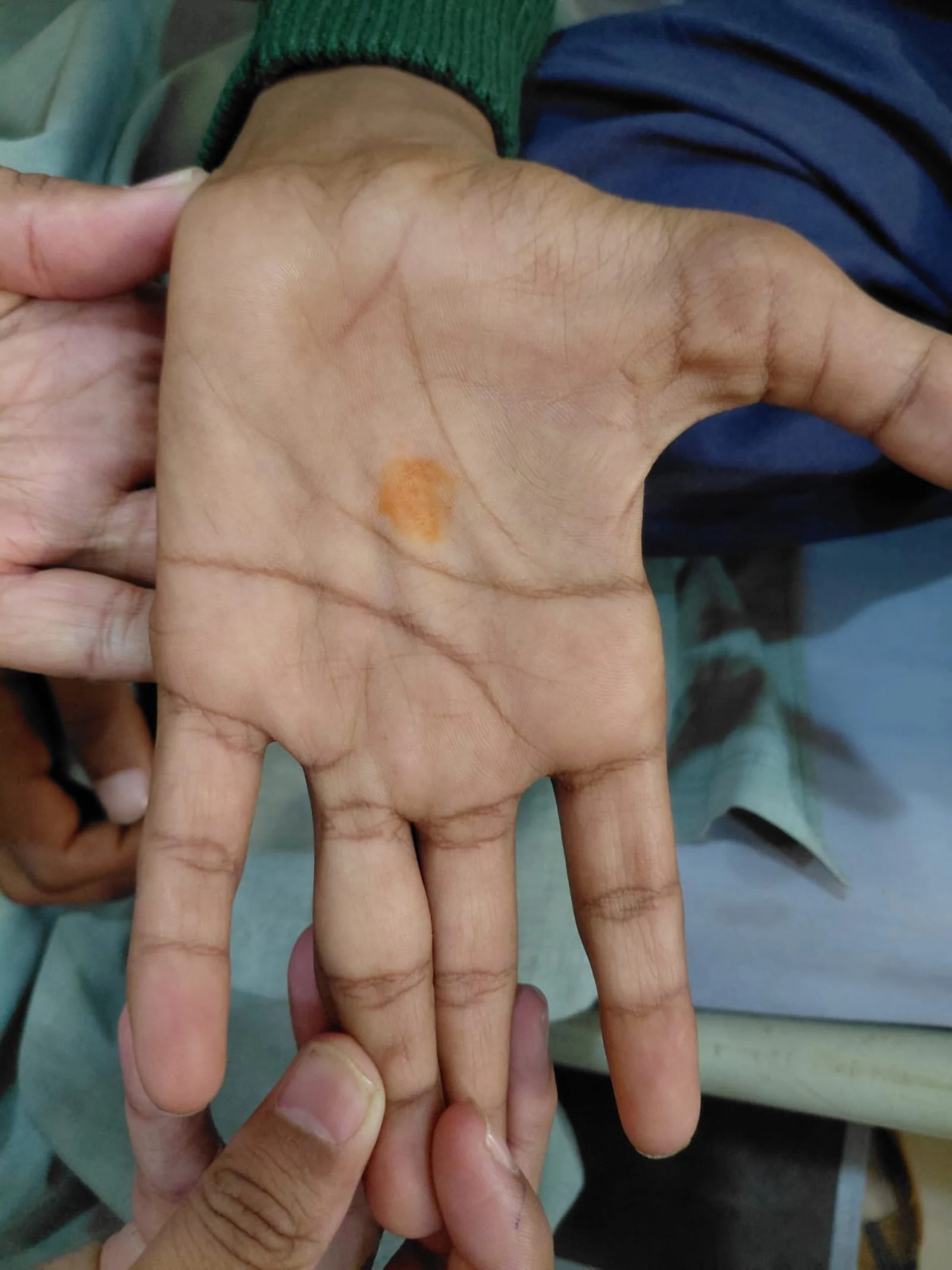
Clinical photograph of the left hand demonstrating marked thenar atrophy. Clinical photograph of the left hand showing prominent wasting of the thenar eminence.

## 4. Diagnostic assessment

The diagnostic assessment was a multi-tiered process aimed at ruling out mimicking conditions. Audiometry confirmed profound bilateral sensorineural hearing loss. Nerve conduction studies revealed a pure motor-predominant axonal neuropathy with reduced compound muscle action potential amplitudes and normal conduction velocities, consistent with a motor neuronopathy. Electromyography showed chronic denervation with fibrillation potentials and positive sharp waves in bulbar, cervical, and lumbosacral myotomes. Crucially, repetitive nerve stimulation at 3 Hz showed no significant decrement, effectively ruling out a neuromuscular junction disorder. MRI of the brain and cervical spine was normal, with no evidence of demyelination or structural brainstem lesion. Serum creatine kinase levels were normal. A comprehensive metabolic screen, including plasma amino acids, urine organic acids, and acylcarnitine profile, was unremarkable a common finding in RTD3.

The primary diagnostic challenge was the normalcy of all these tests, which placed the clinician in a dilemma between the strongly suggestive clinical phenotype and the unrevealing ancillary investigations. The main differential diagnoses included juvenile amyotrophic lateral sclerosis (ALS), Fazio-Londe syndrome (a childhood-onset pontobulbar palsy without deafness), and mitochondrial cytopathies. The normal genetic panels for mitochondrial and hereditary myopathies/dystrophies initially seemed to lower the probability of a neurometabolic disorder. However, the pathognomonic combination of profound deafness, tongue atrophy, a moth-eaten hand (split-hand sign), and the family history of 2 sibling deaths from “respiratory failure” in their twenties was overwhelmingly suggestive of BVVL syndrome. The definitive diagnosis was established by targeted Sanger sequencing of the *SLC52A2* and *SLC52A3* genes, which revealed a homozygous pathogenic frameshift mutation in *SLC52A3*.

## 5. Therapeutic intervention

The primary therapeutic intervention was the immediate initiation of high-dose oral riboflavin (vitamin B2) at a dosage of 40 mg/kg/day divided into 3 doses. This pharmacologic therapy is the sole evidence-based treatment that directly targets the pathophysiology of the disorder by circumventing the defective transporter and restoring intracellular flavin levels through passive diffusion at supraphysiologic concentrations. The intervention was started empirically on Day 5 of admission, prior to the return of the confirmatory genetic test, due to the rapid respiratory decline and the strong clinical index of suspicion. No other pharmacological agents were initiated. Supportive interventions were also critical and included the insertion of a nasogastric tube for safe nutritional and medication delivery and initiation of noninvasive bi-level positive airway pressure ventilation at night to manage hypercapnic respiratory failure. No changes in the riboflavin dose were required; the excellent clinical response guided us to continue the same regimen. The patient was transitioned from an inpatient neuro-intensive care setting to a rehabilitation facility over 10 days.

## 6. Follow-up and outcomes

Clinician-assessed outcomes were dramatic. Within 5 days of initiating riboflavin, the patient reported a significant subjective improvement in his breathing. Objectively, his forced vital capacity measured by bedside spirometry improved from 35% of predicted at baseline to 50% of predicted within 1 week. Limb muscle strength, especially in the hands, improved from Medical Research Council 4–/5 to 4/5 bilaterally, allowing him to regain the ability to self-feed and use his phone. At the 8-week follow-up, the patient had gained 3 kg, and his dysphagia had stabilized such that he could safely tolerate a modified oral diet of soft foods, though the nasogastric tube was kept for supplemental nocturnal feeding. Repeat nerve conduction studies at the 6-month follow-up showed that the previously observed decline in compound muscle action potential amplitudes had completely arrested, suggesting a halt to axonal degeneration. The most important outcome was survival and the prevention of progression to the fatal respiratory failure that had claimed his siblings.

Intervention adherence and tolerability were excellent. Riboflavin is remarkably well-tolerated, with the only notable adverse effect being a benign, temporary yellow-orange discoloration of the urine, which the patient was counseled about. No nausea, diarrhea, or hepatotoxicity was noted. The urine discoloration actually served as a simple, consistent, and patient-reported marker of compliance.

## 7. Discussion

This case highlights both the diagnostic challenges and the therapeutic importance of recognizing riboflavin transporter deficiency in a young patient presenting with progressive neurological disease. The combination of sensorineural deafness, pontobulbar dysfunction, and peripheral motor-predominant neuropathy represents a characteristic phenotype of BVVL/ riboflavin transporter deficiency and should prompt consideration of an underlying riboflavin transporter defect.^[1,2]^ In the present patient, profound hearing loss, tongue atrophy with fasciculations, facial weakness, distal muscle wasting, depressed reflexes, and progressive respiratory compromise provided a coherent clinical pattern despite otherwise unrevealing investigations.

The motor neuronopathy and marked hand-muscle wasting can lead to diagnostic confusion with juvenile ALS or other hereditary motor neuropathies. Late-onset riboflavin transporter deficiency has specifically been described as a treatable mimic of several motor neuropathy phenotypes.^[4]^ Neuropathological and clinical observations in BVVL similarly demonstrate extensive involvement of motor neuronal systems and cranial motor pathways, providing a biological explanation for this clinical overlap.^[5]^ The coexistence of progressive sensorineural deafness is therefore an especially important diagnostic clue when a young patient presents with an ALS -like or motor-neuron-predominant syndrome.

Another important lesson from this case is that negative broad genetic or metabolic testing should not override a strongly characteristic phenotype. Commercial or disease-specific genetic panels vary substantially in gene content, and *SLC52A2* and *SLC52A3* may not necessarily be captured when testing is directed primarily toward mitochondrial disease, hereditary myopathy, or more conventional motor neuron disorders. When the clinical combination of hearing impairment, bulbar involvement, neuropathy, and respiratory weakness is present, targeted testing for riboflavin transporter deficiency or a sufficiently comprehensive genomic approach should therefore be considered.

The available literature demonstrates substantial phenotypic overlap between *SLC52A2*- and *SLC52A3*-related riboflavin transporter deficiencies. *SLC52A2*-associated disease frequently presents earlier and may involve prominent sensory neuropathy, sensory ataxia, and optic abnormalities, whereas later-onset pontobulbar and motor-neuron-predominant phenotypes have particularly been reported with *SLC52A3*-related disease.^[1,2,4]^ Nevertheless, considerable phenotypic variability exists, and genotype alone does not fully predict the age of onset, rate of progression, or severity of neurological involvement.

The therapeutic implications are particularly important because riboflavin transporter deficiency represents one of the uncommon hereditary neuronopathies for which a relatively simple metabolic treatment may alter disease progression. Foley et al demonstrated substantial clinical and biochemical improvement following high-dose riboflavin therapy in genetically confirmed riboflavin transporter deficiency, providing important evidence that neurological deterioration may be partially reversible when treatment is initiated before extensive irreversible neuronal loss has occurred.^[6]^ Although that landmark work focused particularly on RFVT2-related disease, subsequent clinical experience has supported riboflavin responsiveness across the broader riboflavin transporter deficiency spectrum.

The optimal riboflavin dose has not been established through randomized controlled trials, and reported therapeutic regimens vary considerably. Clinical series and case reports have used doses ranging from approximately 10 to 20 mg/kg/day to substantially higher regimens, particularly in patients with severe neurological or respiratory manifestations.^[6,7]^ High-dose treatment is generally well tolerated, allowing clinicians to individualize therapy according to disease severity, clinical response, and tolerability. In the present patient, 40 mg/kg/day was selected because of rapidly progressive respiratory compromise and was followed by rapid improvement without clinically significant adverse effects.

The biological rationale for high-dose riboflavin therapy is also compelling. Riboflavin is the precursor of flavin mononucleotide and flavin adenine dinucleotide, which are required by numerous flavoproteins involved in mitochondrial oxidative phosphorylation and other critical cellular pathways. Defective riboflavin transport can therefore produce secondary mitochondrial dysfunction and impaired cellular energy metabolism, particularly affecting metabolically vulnerable neural tissues.^[2,8]^ Administration of riboflavin at pharmacological doses may increase intracellular availability through residual transporter activity or alternative uptake mechanisms, thereby improving flavin-dependent mitochondrial function.^[8]^

The temporal relationship between treatment initiation and clinical improvement in this patient further supports a therapeutic effect. Before riboflavin administration, the patient demonstrated progressive respiratory deterioration, worsening bulbar dysfunction, and limb weakness against a family background in which 2 siblings had experienced apparently similar fatal illnesses. Within several days of commencing high-dose riboflavin, respiratory effort improved, followed by measurable improvement in forced vital capacity and limb strength. Subsequent weight gain, stabilization of swallowing, and absence of further electrophysiological deterioration over 6 months provided additional evidence of sustained clinical stabilization.

Nevertheless, the response in a single patient cannot establish treatment efficacy independently of the broader literature. Spontaneous fluctuation, supportive respiratory management, improved nutrition, and other aspects of multidisciplinary care may have contributed to some observed improvement. The particularly rapid temporal association with riboflavin initiation, the biologically plausible mechanism, the genetically confirmed diagnosis, and the previously documented treatment responsiveness of riboflavin transporter deficiencies together make a clinically meaningful treatment effect highly plausible.^[1,6–8]^

The most important clinical implication is therefore early recognition. BVVL/RTD3 should be considered in any young patient with progressive sensorineural hearing loss accompanied by bulbar dysfunction, motor or sensorimotor neuropathy, muscle wasting, or unexplained respiratory weakness. A suggestive family history or consanguinity should further increase suspicion, but its absence should not exclude the diagnosis. Targeted genetic testing for *SLC52A2* and *SLC52A3* should be pursued promptly when the phenotype is compatible.

Because neurological injury may become irreversible with prolonged disease duration, treatment should not necessarily be postponed until molecular confirmation when clinical suspicion is high and the patient is deteriorating. Riboflavin is inexpensive, widely available, and generally well tolerated, whereas delayed treatment may allow further axonal loss and respiratory compromise.^[1,6,7]^ The present case illustrates how recognition of a distinctive clinical phenotype and early initiation of high-dose riboflavin can transform the trajectory of an otherwise progressive and potentially fatal neurological disorder.

## Reporting guideline

This case report was prepared in accordance with the CARE (CAse REport) reporting guidelines.

## Author contributions

**Data curation:** Usama Saleem.

**Formal analysis:** Usama Saleem.

**Supervision:** Muhammad Talha.

**Visualization:** Muhammad Talha.

**Resources:** Labeeba Abdul Ghafoor.

**Validation:** Labeeba Abdul Ghafoor.

**Methodology:** Mukesh Kumar Sharma.

**Writing – original draft:** Aasim Ali, Armaghana Abdullah.

**Writing – review & editing:** Hafiz Muhammad Yousaf Masood.

## References

[1] Jaeger B Bosch AM. Clinical presentation and outcome of riboflavin transporter deficiency: mini review after five years of experience. J Inherit Metab Dis. 2016;39:559–64. 10.1007/s10545-016-9924-2 PMC4920840 26973221

[2] O’Callaghan B Bosch AM Houlden H. An update on the genetics, clinical presentation, and pathomechanisms of human riboflavin transporter deficiency. J Inherit Metab Dis. 2019;42:598–607. 10.1002/jimd.12053 30793323

[3] Ciccolella M Catteruccia M Benedetti S. Brown-Vialetto-van Laere and Fazio-Londe overlap syndromes: a clinical, biochemical and genetic study. Neuromuscul Disord. 2012;22:1075–82. 10.1016/j.nmd.2012.05.007 22824638

[4] Carreau C Benoit C Ahle G. Late-onset riboflavin transporter deficiency: a treatable mimic of various motor neuropathy aetiologies. J Neurol Neurosurg Psychiatry. 2020;92:27–35. 10.1136/jnnp-2020-323304 33087424

[5] Malafronte P Clark HB Castaneda-Sanchez I Raisanen J Hatanpaa KJ. Brown-Vialetto-Van Laere syndrome: clinical and neuropathologic findings with immunohistochemistry for C20orf54 in three affected patients. Pediatr Dev Pathol. 2013;16:364–71. 10.2350/13-02-1299-CR.1 23688382

[6] Foley AR Menezes MP Pandraud A. Treatable childhood neuronopathy caused by mutations in riboflavin transporter RFVT2. Brain. 2014;137(Pt 1):44–56. 10.1093/brain/awt315 PMC3891447 24253200

[7] Garg M Kulkarni SD Hegde AU Shah KN. Riboflavin treatment in genetically proven Brown-Vialetto-Van Laere Syndrome. J Pediatr Neurosci. 2018;13:471–3. 10.4103/JPN.JPN_131_17 PMC6413615 30937093

[8] Udhayabanu T Manole A Rajeshwari M Varalakshmi P Houlden H Ashokkumar B. Riboflavin responsive mitochondrial dysfunction in neurodegenerative diseases. J Clin Med. 2017;6:52. 10.3390/jcm6050052 PMC5447943 28475111

